# Validity of Sequential Organ Failure Assessment and Quick Sequential Organ Failure Assessment in Assessing Mortality Rate in the Intensive Care Unit With or Without Sepsis

**DOI:** 10.7759/cureus.11071

**Published:** 2020-10-20

**Authors:** Maleeha Ali Basham, Hassan Ali Ghumro, Muhammad Usman Shah Syed, Sumayyah Saeed, Syed Annas Pervez, Umar Farooque, Naresh Kumar, Zainab Imtiaz, Muhsana Sajjad, Aisha Jamal, Iqra Aslam Siddiqui, Farha Idris

**Affiliations:** 1 Internal Medicine, Dow University of Health Sciences, Karachi, PAK; 2 Internal Medicine, Civil Hospital Karachi, Dow University of Health Sciences, Karachi, PAK; 3 Neurology, Dow University of Health Sciences, Karachi, PAK; 4 Medicine, Dow University of Health Sciences, Karachi, PAK; 5 Internal Medicine, Lahore Medical and Dental College, Lahore, PAK; 6 Surgery, Civil Hospital Karachi, Dow University of Health Sciences, Karachi, PAK

**Keywords:** sofa, qsofa, sequential organ failure assessment, quick sequential organ failure assessment, validity, mortality rate, intensive care unit, sepsis, humans, septic shock

## Abstract

Introduction

Sepsis and septic shock (sepsis-induced hypotension not improved by adequate fluid resuscitation) are among the most common reasons for admission to an intensive care unit (ICU) and display high mortality rates. Different scoring systems are used to diagnose and predict the mortality of patients having sepsis. This study aims to validate the prognostic accuracy of Sequential Organ Failure Assessment (SOFA) and Quick Sequential Organ Failure Assessment (qSOFA) in determining the mortality of both septic and non-septic patients.

Materials and methods

This retrospective cohort study was conducted in May 2018 in the Surgical Intensive Care Unit (SICU) of a tertiary care hospital in Karachi, Pakistan. Past 200 patient records, from January 2018 to April 2018, were examined, and 20 records were discarded due to insufficient data. Sufficient observational data were collected, which was used to assess the validity of the SOFA and qSOFA in determining the mortality rate of sepsis. A comparison of the two modalities was made.

Results

Out of the 200 patients, 180 were enrolled. Data from their entire ICU stay were used to calculate their initial, highest, and mean SOFA and qSOFA. Mean SOFA score up to nine correlated with a mortality rate of up to <79%, while scores 10 and above predicted a 100% mortality rate. A mean qSOFA score of three predicted a 67% mortality rate. Univariate logistic analysis performed with odds ratio showed that the mean qSOFA score was in comparison more closely able to predict mortality, followed by mean SOFA score (p values < 0.01).

Conclusions

This study concluded that both SOFA and qSOFA scores are good predictors of mortality. However, qSOFA is more closely accurate in predicting mortality than SOFA. But further analysis with larger sample size for a longer duration as well as the application of these scores in the emergency departments and general wards can prove the precision of this study.

## Introduction

There are many complexities in the medical interventions performed in intensive care units (ICUs), which explain the high mortality rates observed [1]. Sepsis is a common cause of admission and death in ICUs across the globe [2]. It is a dysregulated response to infection, which can be identified by using the Systemic Inflammatory Response Syndrome (SIRS) criteria. In addition, severe sepsis is marked by organ dysfunction. Septic shock is a result of hypotension caused by sepsis, which persists even after fluid resuscitation, causing decreased tissue perfusion. [3]. According to the World Health Organization (WHO), 30 million people worldwide suffer from sepsis every year, three million of these are newborns, and 1.2 million are children. Five to six of these people are unable to survive [4]. Unfortunately, there is insufficient data on sepsis-related outcomes and deaths in the ICUs of Pakistan [5].

Clinicians make use of several scoring systems to diagnose sepsis and predict its mortality. These include Acute Physiological and Chronic Health Evaluation (APACHE), Mortality Probability Model (MPM), and Simplified Acute Physiological Score (SAPS), which predict mortality based on data from the first day of ICU stay. Sequential Organ Failure Assessment (SOFA) and Quick Sequential Organ Failure Assessment (qSOFA) are dynamic scores that predict mortality using data from the entire ICU stay [6].

Out of the precedent models, we adopted the SOFA and qSOFA to determine the patient’s organ function and rate of failure or mortality. SOFA score helps to monitor the patient’s health status during their stay in an ICU. It is used to ascertain organ function and its rate of failure. Contrary to its counterparts, SOFA scoring focuses more on organ dysfunction and morbidity than predicting the mortality of the patient [7]. It also assesses the function of the patient’s nervous, cardiovascular, and respiratory systems as well as their liver function and coagulation, and renal function, with a minimum score of zero representing no organ dysfunction. On the other hand, qSOFA was introduced as a quick and simplified version of the SOFA to help identify critically ill patients with sepsis who were high-risk patients needing higher level care. It looks at the Glasgow Coma Scale (GCS) of <15 to determine altered mental status, systolic blood pressure of 100 mm Hg or less, and respiratory rate of 22/min or greater [3].

Our main objective was to assess the validity of SOFA and qSOFA in determining the mortality of ICU patients, irrespective of their cause of admission, in a public sector teaching hospital of Karachi, Pakistan. Our study compared the qSOFA and SOFA scores in predicting the mortality using the initial, highest, and mean SOFA and qSOFA scores.

The use of SOFA and qSOFA scores in our study is authenticated by research reporting that the other criteria have an inappropriate predictive capacity. These scores, namely APACHE I and II, had been devised decades ago. APACHE II and III predict outcomes within only 24 hours of an ICU stay [8]. A retrospective cohort analysis comparing the predictive potential of SOFA, qSOFA, and SIRS managed to demonstrate the poor accuracy of the SIRS score compared to SOFA and qSOFA [4]. Kaukonen and colleagues in their retrospective study, conducted from 2000 to 2013 in 172 ICU units of Australia and New Zealand, also elaborated that the SIRS criteria failed to define a transition point in the risk of death [9]. APACHE III model was described to be a deteriorated model in a 10-year study in New Zealand and Australia [10]. Another retrospective cohort study at the University of California, San Francisco, compared the University Health Consortium Expected Probability of Mortality (UHC-EPM) and MPM to anticipate death and mentioned that MPM consistently overpredicted mortality in their patients [11].

## Materials and methods

Study design and sampling

This retrospective cohort study was conducted in May 2018. Four months of data were collected, from January 2018 to April 2018, from a Surgical Intensive Care Unit (SICU) of a tertiary care hospital in Karachi, Pakistan. The study was approved by the Institutional Review Board (IRB) of the Dow University of Health Sciences. The hospital has an easily accessible locality for patients from different socioeconomic backgrounds. Written permission was obtained from the Head of Department of the SICU to access the patient records. The patients who fulfilled the inclusion criteria included all patients from nine to 82 years old with complete data and laboratory investigations admitted to the SICU department. Exclusion criteria involved all records with incomplete data, not meeting the SOFA and qSOFA criteria.

Data collection

Access to past patient records was granted, and data were collected from 200 records, out of which 20 were excluded due to incomplete data. Patient intervention was not required; hence, informed consent was waived. The data collected had three domains: the first domain included background characteristics including the total number of patients, age, and gender. The second domain included the length of ICU stay and the current diagnosis. The third domain consisted of known co-morbidities, patient assessment, and the total number of deaths. SOFA and qSOFA scores were assessed starting from the time of admission up to the time of discharge or death. The questionnaire was reviewed by a senior surgeon of the hospital to ensure that it covered all the major aspects of patients’ information. A pilot study was conducted on 30 patients to ensure the validity of the questionnaire. It has not been published anywhere.

Data analysis

Data were analyzed using Statistical Package for Social Sciences (SPSS) version 23.0 (Armonk, NY: IBM Corp). The GCS was used to evaluate the neurological status in all the patients, while in sedated patients, the assumed GCS scale was utilized. During the calculation of the SOFA and qSOFA scores, only the worst values for each status in 24 hours were used. During the ICU stay for all patients, the mean SOFA and the mean qSOFA scores were calculated as the sum of all daily SOFA and qSOFA scores divided by the length of ICU stay, respectively. The initial and the highest SOFA and qSOFA scores were calculated as well. For a single missing value, a replacement value from the mean of results preceding and following the missing value was calculated. Univariate regression analysis was done, and an odds ratio with a 95% confidence interval was determined. ICU outcome was taken as the dependent variable. Continuous variables such as initial, mean, and highest scores of SOFA and qSOFA were represented using bar charts. p value ≤ 0.05 was considered significant.

## Results

A total of 180 patients were enrolled in this study. The distribution of the sample in the group "Surgical Sepsis" was 17.2%, and "Surgical Non-Sepsis" was 62%, while the group "Non-Surgical Sepsis" was 2%, and "Non-Surgical Non-Sepsis" was 19.4%. These demographics of eligible subjects are shown in Table 1.

**Table 1:** Demographics of the study population. ICU, Intensive care unit; PPH, postpartum hemorrhage; Ca, cancer.

Demographic variables		Values
Total number of patients		180
Age (years)	Mean	33
	Standard deviation	13.3
	Range	9-82
Gender	Male	70
	Female	110
Length of ICU stay (days)	Mean	1.1
	Standard deviation	0.34
	1-3 days (%)	86
	>3 days (%)	14
Co-morbidities (%)	Hypertension	4
	Hepatitis B/Hepatitis C	5
	None	90
Diagnoses (%)	Eclampsia/PPH	20
	Peritonitis	7
	Ca cheek	7
	Sepsis	4
	Others	62
Patient assessment (%)	Surgical sepsis	17.2
	Surgical non-sepsis	62
	Non-surgical sepsis	2
	Non-surgical non-sepsis	19.4
Total number of deaths		57

**Figure 1:**
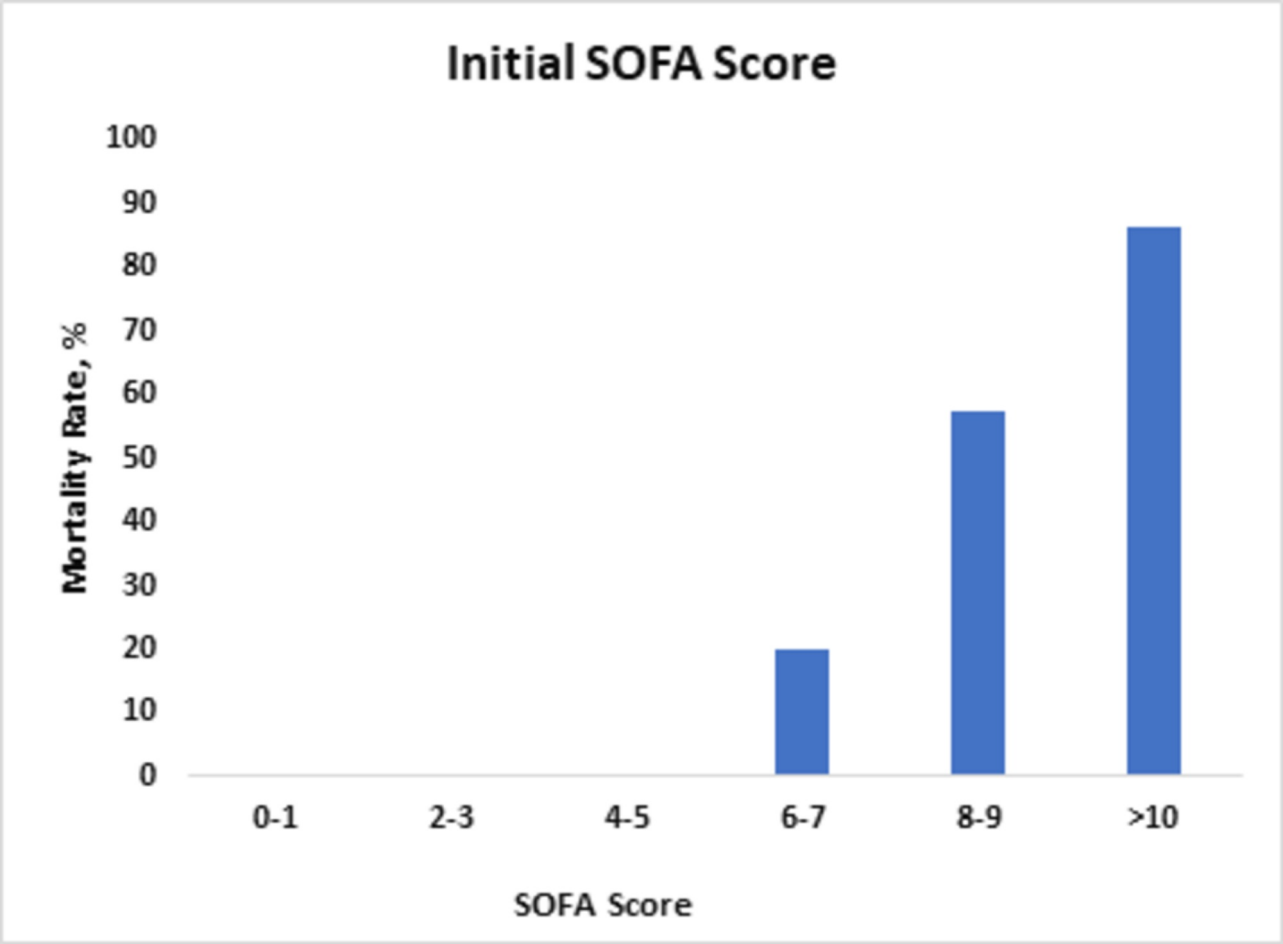
Mortality rates in relation to changes in initial SOFA score. SOFA, Sequential Organ Failure Assessment.

An initial SOFA score of up to seven predicted mortality of <20%, while an initial SOFA score of >10 predicted a mortality rate of 86% (Figure 1).

**Figure 2:**
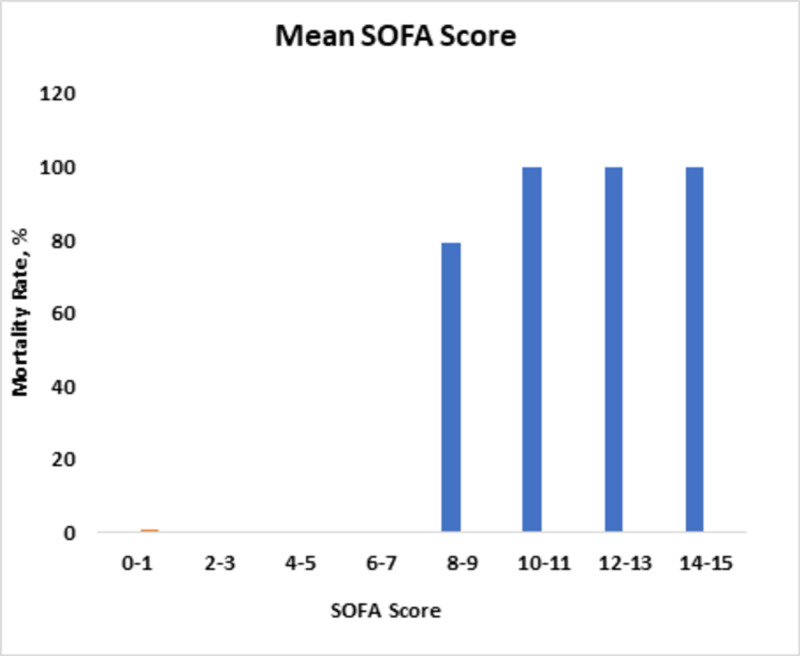
Mortality rates in relation to changes in mean SOFA score. SOFA, Sequential Organ Failure Assessment.

The mean SOFA score also correlated with mortality. Mean SOFA scores up to nine correlated with a mortality rate of up to <79%, while scores 10 and above predicted a 100% mortality rate (Figure 2).

**Figure 3:**
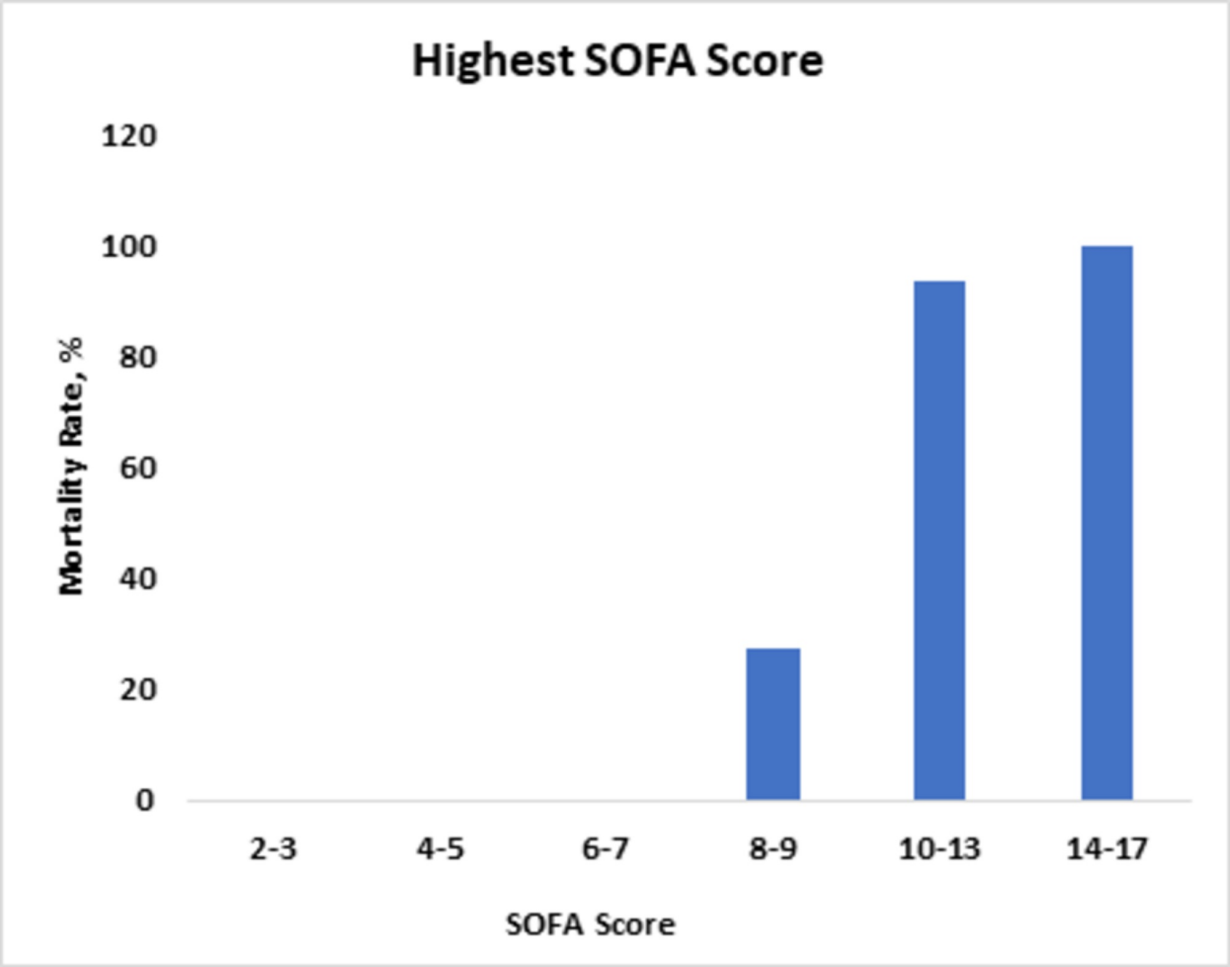
Mortality rates in relation to changes in highest SOFA score. SOFA, Sequential Organ Failure Assessment.

The highest score of nine correlated with a mortality rate of 27%. Scores > 10 correlated with a mortality rate of >93% (Figure 3).

**Figure 4:**
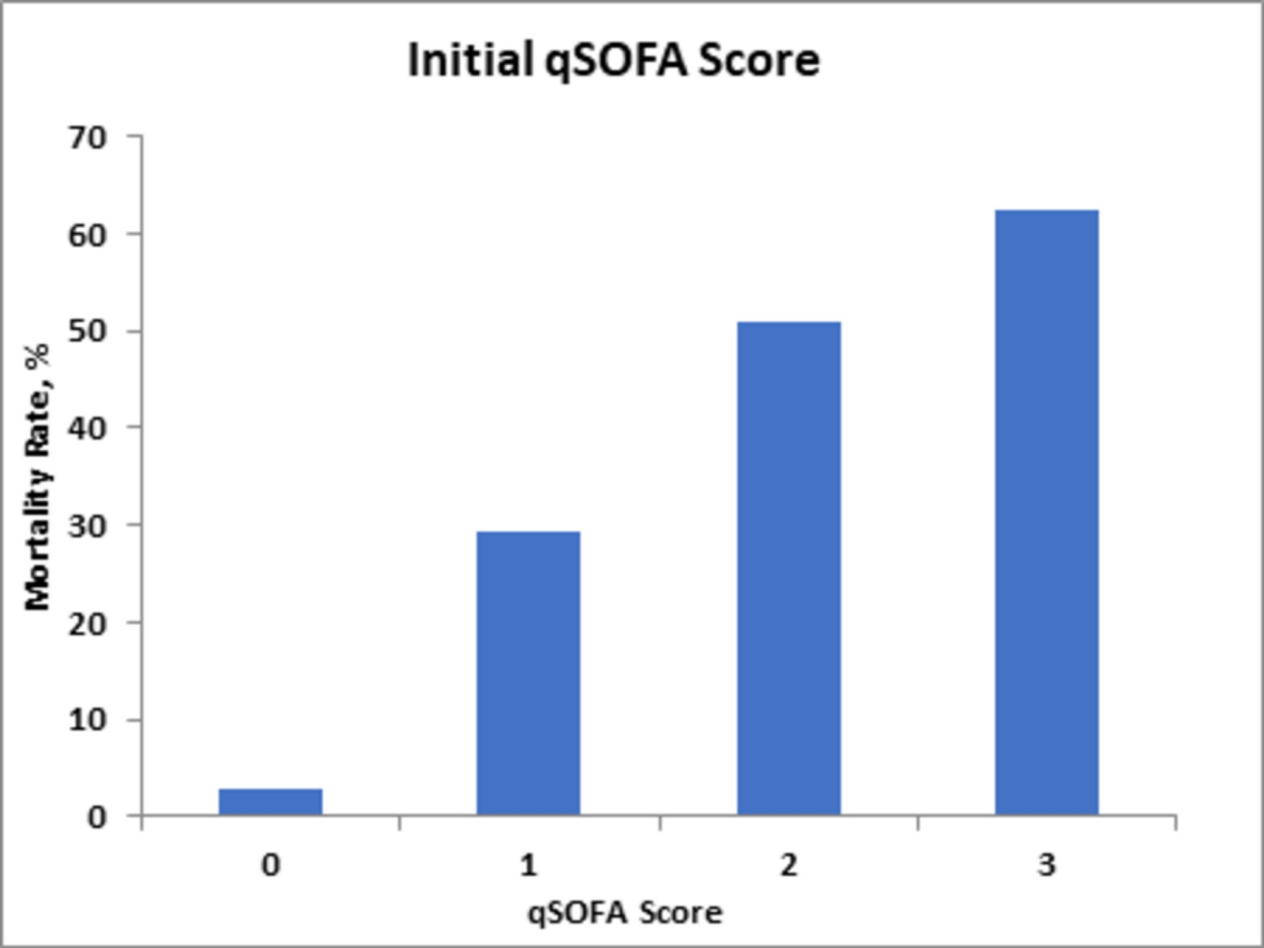
Mortality rates in relation to changes in initial qSOFA score. qSOFA, Quick Sequential Organ Failure Assessment.

**Figure 5:**
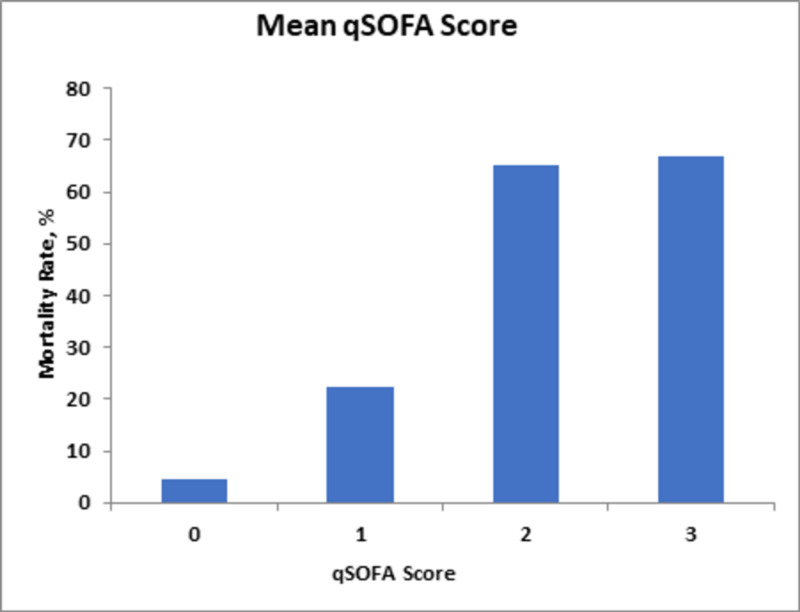
Mortality rates in relation to changes in mean qSOFA score. qSOFA, Quick Sequential Organ Failure Assessment.

**Figure 6:**
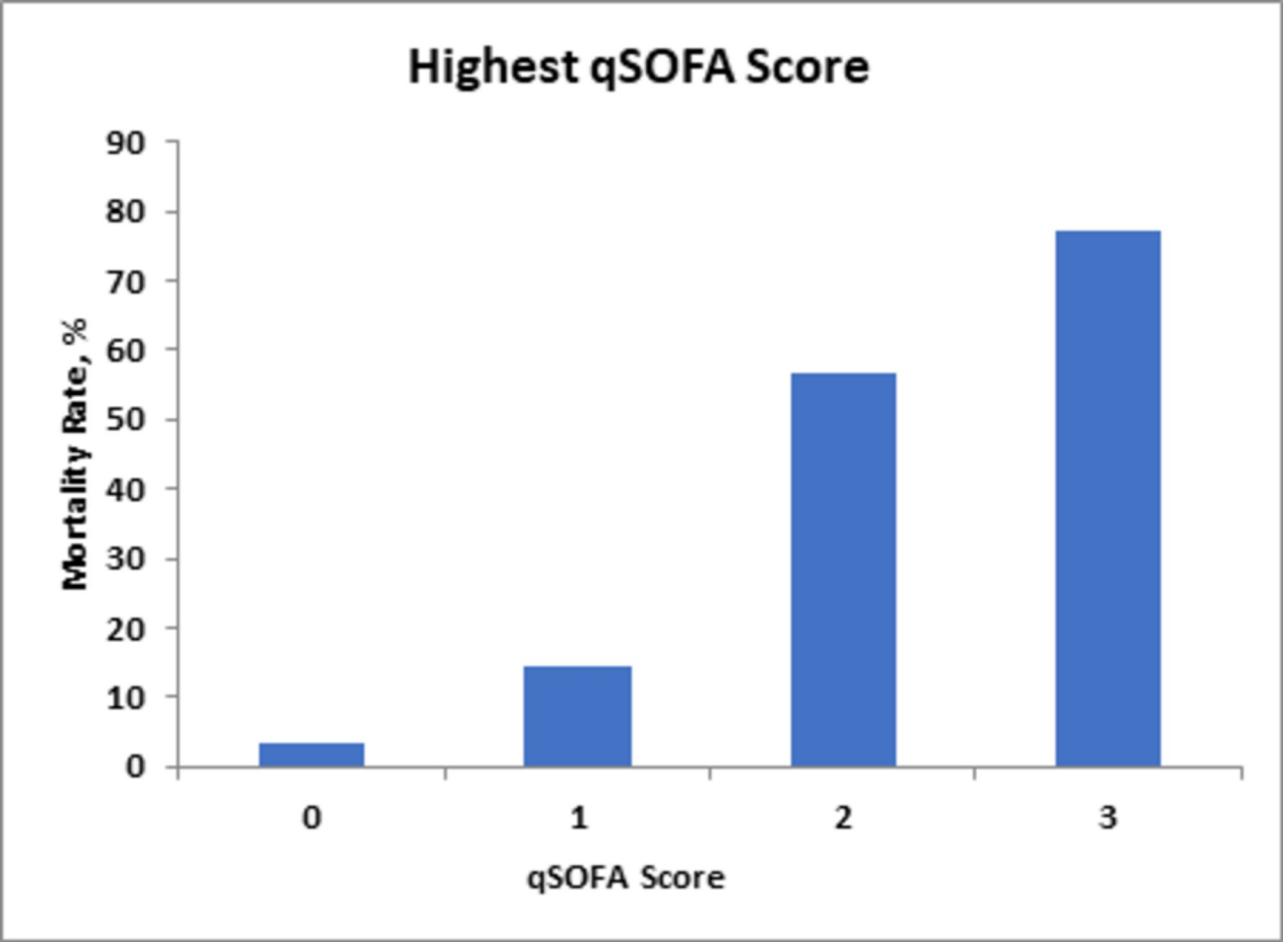
Mortality rates in relation to changes in highest qSOFA score. qSOFA, Quick Sequential Organ Failure Assessment.

On the other hand, the initial qSOFA and mean qSOFA scores correlated less with the mortality rate; scores of two and three predicted 62% and 67% mortality rates, respectively, as compared to the highest qSOFA score, with scores of three correlating to mortality rates of 77% (Figures 4-6).

Using univariate logistic analysis, the odds ratio showed that the mean qSOFA score predicted mortality most closely, followed by the mean SOFA score (p values < 0.01), as shown in Table 2.

**Table 2:** Univariate logistic regression analysis of SOFA and qSOFA as predictors of mortality. SOFA, Sequential Organ Failure Assessment; qSOFA, Quick Sequential Organ Failure Assessment.

Variables	Coefficient mean (SE)	Odds ratio (95% confidence interval)	p value
Initial SOFA score	0.965 (0.156)	2.626	<0.01
Mean SOFA score	1.926 (0.336)	7.738	<0.01
Highest SOFA score	1.637 (0.306)	5.142	<0.01
Initial qSOFA score	1.149 (0.244)	3.154	<0.01
Mean qSOFA score	2.173 (0.341)	8.784	<0.01
Highest qSOFA score	1.737 (0.293)	5.680	<0.01

## Discussion

Recently used scoring models, including APACHE ll, APACHE lll, APACHE lV, SAPSll, MPM ll, SOFA, and qSOFA, have been devised to predict the mortality rate in the ICU settings [12-14]. SOFA and qSOFA criteria were adopted in this study to determine organ functionality and their rate of failure or mortality of the patients. The use of SOFA and qSOFA in both ICU and non-ICU settings has been validated by a prospective observational cohort study in 2017 [15].

SOFA has a good anticipating potential to determine ICU mortality. This was demonstrated by a research conducted in 182 Australian and New Zealand ICUs, where an increase in the SOFA score from baseline of two or more was determined to have patient mortality of 90.1% [4]. Our statistical analysis also showed a directly proportional relationship between SOFA score and mortality rate, with a p-value of <0.01. Another research in 2001, at University Hospital of Belgium, authenticated the scoring credibility of SOFA where the initial SOFA score of nine or more depicted mortality of <33%, and an initial SOFA score of >11 determined mortality of 95% [8]. The highest SOFA score was also evaluated, and a score of 11 or more correlated with >80% mortality. Likewise, in our study, an initial SOFA score of up to eight predicted mortality of 20%, and an increase in initial SOFA score of up to 10 or more correlated with >80% mortality. The highest SOFA score of eight predicted mortality of >30%, and an increase in the score up to 10 predicted >90% mortality. Another study validated the mean SOFA score and correlated it with mortality. In this study, the mean SOFA greater than five predicted the mortality rate of 100%. Using univariate logistic analysis, we determined that the mean SOFA score of up to nine correlated with 79% mortality. We saw that the mortality rate became 100% as the score became >10. The rationality behind the use of SOFA in septic as well as non-septic settings has also been confirmed [4,14]. 

The qSOFA score has been used as a measure of outcome predictability in ICU admissions with or without sepsis. A retrospective study performed in 2014-2015 signified that qSOFA scores correspond well with mortality, showing a mortality rate of 27.4% with a score of three in ICU admissions [16]. In our study, the highest qSOFA score of greater than three correlated with 77% mortality. Using our study, we have demonstrated that the variable highest qSOFA score has a significant association with an increased mortality rate with a p value of <0.01 (Table 2). A comparative cohort prospective observational study using qSOFA and SOFA scores, done at an emergency department of a low-income country using 760 subjects, determined that the sensitivity and predictability of qSOFA in predicting mortality were 96% and 87%, respectively. This was assessed by area under the receiver operating curve (AUROC) [15]. Our analysis via univariate logistic regression demonstrated a mean qSOFA score of three showing a 67% mortality rate, whereas the initial qSOFA score of greater than three shows a 63% mortality rate. 

SOFA and qSOFA scores have been established to be good predictors of mortality above. Further results of our data analysis and the difference in odds ratio directed our study in favor of qSOFA (Table 2). Yet, some studies have contradicted the validity of qSOFA. A Norwegian observational study carried out in the year 2012 signified that a qSOFA score of greater than two has a sensitivity of 32% and specificity of 98% [17]. A 12-month retrospective study conducted by Tusgul and colleagues also denied the prognostic potential of qSOFA score on the basis of hospital stay [18]. However, a number of other studies have verified its prognostic superiority. A retrospective study conducted between July 2015 and December 2016, at Sanglah General Hospital, described a qSOFA score greater than three depicting a specificity of 94.95% and sensitivity of 67.74%. Therefore, we conclude that qSOFA is a validated mortality prediction model [6]. Furthermore, a study conducted at a low-income setup by Baig and colleagues also determined the discriminative capacity of qSOFA score to be sufficient [15]. According to our research, qSOFA was understood to be a better measure of mortality on the basis of its parameters and its advantage to be easily accessible and less time-consuming.

This study had a few limitations. It was carried out in only one SICU, which limited our sample size. A study like this requires a multidisciplinary approach. Since only two scoring systems, i.e., SOFA and qSOFA were used, other scoring systems and their comparisons to SOFA and qSOFA are also necessary to validate the true prognostic capacity of these scoring systems in the ICU settings. Additionally, the data of the qSOFA parameters were only taken from ICU patients, though it is usually carried out in non-ICU patients. The estimation of its predictive potential between ICU and non-ICU patients was not validated.

## Conclusions

There is a correlation between the SOFA and qSOFA scores and the risk of mortality. Despite both being adequate prognostic markers of mortality, qSOFA can predict mortality more closely than SOFA in both septic and non-septic patients. The implementation of these scores in the emergency departments and general wards can further prove their roles. More retrospective and prospective studies should also be done at a longer duration in other hospital settings to validate their significance. SOFA and qSOFA have parameters that are mentioned throughout the hospital stay, the significance of which should also be evaluated. The parameters for SOFA are more in number than the parameters required for qSOFA score; therefore, specifying the parameters in both criteria and devising a new prediction model for convenience and better results are crucial.
